# Study of Drug Metabolism by Xanthine Oxidase

**DOI:** 10.3390/ijms13044873

**Published:** 2012-04-18

**Authors:** Jing Zhao, Xiaolin He, Nana Yang, Lizhou Sun, Genxi Li

**Affiliations:** 1 Laboratory of Biosensing Technology, School of Life Sciences, Shanghai University, Shanghai 200444, China; E-Mails: jzhao11@gmail.com (J.Z.); hklhxl@126.com (X.H.); 2 Department of Obstetrics, the First Affiliated Hospital of Nanjing Medical University, Nanjing 210036, China; E-Mail: nanayang210@163.com; 3 Department of Biochemistry and State Key Laboratory of Pharmaceutical Biotechnology, Nanjing University, Nanjing 210093, China

**Keywords:** xanthine oxidase, electrochemistry, drug metabolism, 6-mercaptopurine, quercetin

## Abstract

In this work, we report the studies of drug metabolism by xanthine oxidase (XOD) with electrochemical techniques. Firstly, a pair of stable, well-defined and quasi-reversible oxidation/reduction peaks is obtained with the formal potential at −413.1 mV (*vs.* SCE) after embedding XOD in salmon sperm DNA membrane on the surface of pyrolytic graphite electrode. Then, a new steady peak can be observed at −730 mV (*vs.* SCE) upon the addition of 6-mercaptopurine (6-MP) to the electrochemical system, indicating the metabolism of 6-MP by XOD. Furthermore, the chronoamperometric response shows that the current of the catalytic peak located at −730 mV increases with addition of 6-MP in a concentration-dependent manner, and the increase of the chronoamperometric current can be inhibited by an XOD inhibitor, quercetin. Therefore, our results prove that XOD/DNA modified electrode can be efficiently used to study the metabolism of 6-MP, which may provide a convenient approach for *in vitro* studies on enzyme-catalyzed drug metabolism.

## 1. Introduction

Drug metabolism is a process that transforms drug molecules into polar, water-soluble and excretable metabolites through enzymatically catalyzed reactions [1]. Studies on drug metabolism can provide plenty of information about the metabolic pathway of metabolic intermediate and individual differences in drug response, which may play an important role in drug development. *In vitro* methods, having the advantages of large-scale metabolite assay and assessment of species differences in metabolism, have been widely used for the studies of drug metabolism [2,3]. However, the traditional methods, like mass spectrometry (MS) and high performance liquid chromatography (HPLC), are usually time-consuming and expensive [4,5]. Considering the importance of drug metabolism, more techniques should be developed for the studies of drug metabolism, and an electrochemical technique, which is of small size, quite low cost, easy to operate and capable of continuous measurements, has attracted increasing attention for its application in the studies of drug metabolism. Electrochemical technique with extremely no toxicity can mimic the redox reactions of the biomolecules as well as the drug metabolism in the living organisms *in vitro*, and the electrode can be a favorable electron donor taking place of the conventional electron supply systems. Moreover, electrochemical technique may identify the interaction between drug-metabolizing enzyme and the drug molecules in real time and efficiently monitor both the stable metabolite and the unstable intermediary products in a rapid and sensitive manner, which are much more difficult to realize by the traditional methods. Therefore, electrochemical technique has become a welcome alternative to the traditional methods for *in vitro* drug metabolism studies [6–11].

Xanthine oxidase (XOD) is a metalloprotein that catalyzes the purine catabolism and also of major medical interest as a target of many drugs against several diseases in humans, such as gout, hyperuricaemia and chronic heart failure [12]. Although XOD has been implicated as a key oxidative enzyme to construct electrochemical sensors for the determination of hypoxanthine and xanthine [13–15], no electrochemical investigation has been performed on the studies of the drug metabolism by XOD. In this work, by using a XOD/DNA modified pyrolytic graphite (PG) electrode, we have studied the catalysis of 6-mercaptopurine (6-MP) by XOD with electrochemical techniques. Compared with the traditional studies, our method displays a remarkably improved performance, which is simple, fast and sensitive, so it may be very promising in the fields of clinical medicine and pharmacology in the future.

## 2. Results and Discussion

**Figure 1:**
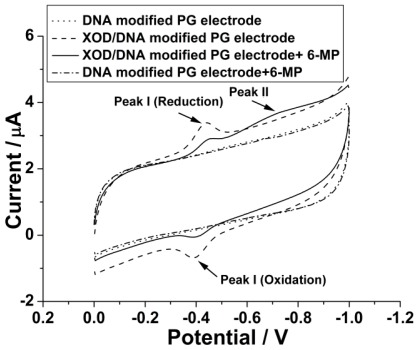
Cyclic voltammograms obtained at DNA and xanthine oxidase (XOD)/DNA modified electrode in the absence and presence of 200 μM 6-MP in 0.1 M pH 7.4 phosphate buffers. Scan rate: 100 mV/s.

Our previous studies have proven that salmon sperm DNA can provide a desirable membrane environment for facilitating the electron transfer between XOD and an electrode [16], so we have firstly examined the XOD/DNA modified PG electrode with cyclic voltammetry (CV). As shown in Figure 1, a pair of well-defined, reversible oxidation/reduction peaks (Peak I) can be observed with the formal potential at −413.1 mV (*vs.* SCE), contributing to the electron transfer between the electro-active center of the immobilized XOD and the electrode. The peak separation of the peak pair is only 51.6 mV at the scan rate of 100 mV/s, which indicates a fast heterogeneous electron transfer process on the XOD/DNA modified electrode. As a comparison, no voltammetric peak can be obtained at the salmon sperm DNA alone modified PG electrode under the same condition.

We have then chosen 6-MP, an anticancer drug for the treatment of acute lymphoblastic leukemia and inflammatory bowel disease [17–19], as the substrate of the enzyme to realize the electrochemical studies of drug metabolism by XOD. It can be observed from Figure 1 that, in the presence of 200 μM 6-MP, a new reduction peak (peak II) appears at −730 mV (*vs.* SCE) on the XOD/DNA modified electrode, while no voltammetric response can be obtained on the DNA alone modified electrode although 6-MP is added in the test solution. So, the appearance of the new peak may be attributed to the metabolism of 6-MP by XOD and the generation of the metabolite, e.g., 6-thiouric acid. Meanwhile, the peak current of XOD (peak I) has a little decrease, which indicates that the binding of 6-MP to XOD may inhibit the electron transfer between XOD and the electrode. Therefore, the CV studies have proven that salmon sperm DNA membrane can not only facilitate the electron transfer between XOD and the electrode, but also well maintain the native activity of XOD, and, moreover, the XOD/DNA modified electrode can be efficiently used to catalyze the metabolism of 6-MP.

**Figure 2:**
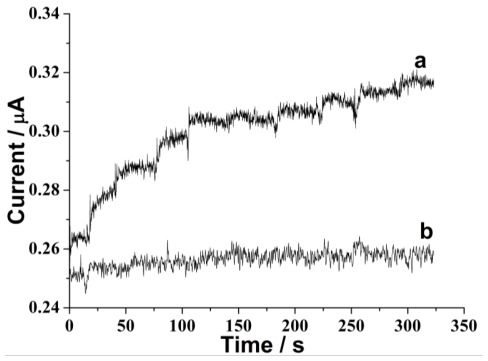
Chronoamperomograms obtained at (**a**) XOD/DNA and (**b**) DNA modified electrode in 0.1 M pH 7.4 phosphate buffers with injecting 200 μM 6-MP every 30 s. The detection potential was −730 mV.

We have further employed a more sensitive electrochemical method chronoamperometry to study the metabolism of 6-MP by XOD. Figure 2 shows the chronoamperometric responses obtained at the XOD/DNA and DNA modified electrode with the continuous addition of 6-MP. It can be observed that on the XOD/DNA modified electrode, the current of the peak located at −730 mV evidently increases with the addition of 6-MP in a concentration-dependent manner, while the current hardly changes on the DNA alone modified electrode. Similar to CV results, this phenomenon indicates that the metabolism of 6-MP can be catalyzed by XOD/DNA modified electrode in a feasible way.

**Figure 3:**
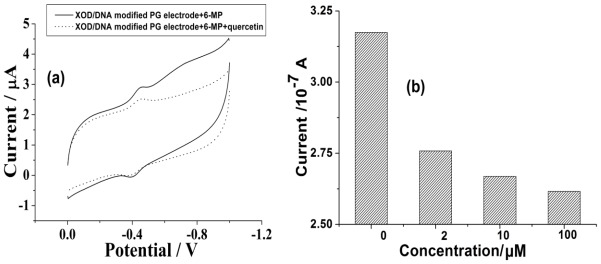
(**a**) Cyclic voltammograms obtained at XOD/DNA modified electrode for 200 μM 6-MP in the absence and presence of 200 μM quercetin, others same as in Figure 1; (**b**) The chronoamperometric currents obtained at the XOD/DNA modified electrode for 2 mM 6-MP in the presence of different concentrations of quercetin (0, 2, 10, 100 μM) in 0.1 M phosphate buffer (pH 7.4).

In order to understand more precisely about the metabolism of 6-MP by XOD, we have further studied the effect of an inhibitor of the enzyme, quercetin, on this metabolism process, which is a major representative of flavonols and a dietary antioxidant proposed as a mechanism for curing the diseases related with XOD [20,21]. Since the metabolism of 6-MP by XOD may be inhibited by quercetin, the peak current of the metabolite located at −730 mV should correspondingly decrease. Figure 3a is the cyclic voltammogram obtained at the XOD/DNA modified electrode with the addition of 200 μM 6-MP in the absence and presence of 200 μM quercetin. It has shown that the new catalytic peak at −730 mV has become indistinguishable in the presence of quercetin, suggesting a significant inhibition of quercetin on the catalytic activity of XOD. Furthermore, we have studied the inhibitory effect with chronoamperometry. As shown in Figure 3b, the current of the catalytic peak for 2 mM 6-MP at −730 mV obtained in the chronoamperomogram does indeed decrease in the presence of quercetin, and the decrease of the current is observed to be stronger with a higher quercetin concentration. So, both the cyclic voltammogram and chronoamperomogram have proven the inhibitory effect of quercetin on the metabolism by XOD, and reconfirmed that the electrochemical technique can be used as a powerful *in vitro* method for the studies of drug metabolism.

## 3. Experimental Section

### 3.1. Reagents

XOD, 6-MP, salmon sperm DNA and quercetin were obtained from Sigma. The solutions were prepared with double-distilled water (18 MΩ·cm), which was purified with a Milli-Q ultrapure water system.

### 3.2. Preparation of XOD/DNA Modified Electrode

The substrate PG electrode was firstly polished using rough and fine sand papers. Then, it was polished to mirror smoothness with aluminum oxide (particle size of about 0.05 μm)/water slurry on silk. Finally, the electrode was thoroughly washed by ultrasonicating in both doubly distilled-water and ethanol for 5 min, respectively. Afterward, a mixture of 10 μL 3.5 U/mL XOD and 10 μL 1.0 mg/mL salmon sperm DNA solution was evenly spread on the surface of the PG electrode. The modified electrode was dried overnight at room temperature in the dark, and was thoroughly rinsed with double-distilled water before use.

### 3.3. Electrochemical Measurements

Electrochemical experiments were performed on a CHI 440a electrochemical analyzer (CH Instrument), using a three-electrode configuration. The reference electrode was a saturated calomel electrode (SCE) and the counter electrode was a platinum electrode. All the test solutions were bubbled thoroughly with high-purity nitrogen through the solution for at least 10 min. A stream of nitrogen was then blown gently across the surface of the solution to maintain the solution anaerobic throughout the experiments.

## 4. Conclusions

In summary, we have successfully demonstrated an electrochemical approach to study the drug metabolism by XOD. While DNA membrane on the PG electrode surface can well maintain the activity of XOD and facilitate the electron transfer process, both voltammetric and chronoamprometric results have proven the metabolism of 6-MP by XOD, which can be further prohibited by the XOD inhibitor, quercetin. Our electrochemical approach has overcome several drawbacks of the traditional methods for *in vitro* studies on drug metabolism, such as its laborious and time-consuming methods. Therefore, this approach may provide a new strategy for *in vitro* studies of drug metabolism that may facilitate drug development and clinical application in the future.
